# Identification of a long non-coding RNA as a novel biomarker and potential therapeutic target for metastatic prostate cancer

**DOI:** 10.18632/oncotarget.1769

**Published:** 2014-02-06

**Authors:** Francesco Crea, Akira Watahiki, Luca Quagliata, Hui Xue, Larissa Pikor, Abhijit Parolia, Yuwei Wang, Dong Lin, Wan L. Lam, William L. Farrar, Takao Isogai, Rudolf Morant, Serenella Castori-Eppenberger, Kim N. Chi, Yuzhuo Wang, Cheryl D. Helgason

**Affiliations:** ^1^ Experimental Therapeutics, BC Cancer Agency Cancer Research Centre, Vancouver BC, Canada; ^2^ The Vancouver prostate Centre, Vancouver General Hospital, Vancouver BC, Canada; ^3^ Molecular Pathology Unit, Institute of Pathology, University Hospital Basel, Switzerland; ^4^ Genetics Unit, Integrative Oncology, BC Cancer Agency Cancer Research Centre, Vancouver BC, Canada; ^5^ Honours Biotechnology, Department of Microbiology and Immunology, University of British Columbia, Vancouver BC, Canada; ^6^ Cancer Stem Cell Section, National Laboratory at Frederick, MD, USA; ^7^ Translational Research Center, Fukushima Medical University, Fukushima, Japan; ^8^ Cancer Center, ZeTuP AG St.Gallen, St.Gallen, Switzerland; ^9^ Medical Oncology, BC Cancer Agency Vancouver Cancer Centre, Vancouver BC, Canada

**Keywords:** long non-coding RNA, prostate cancer, metastasis, androgen receptor, cancer biomarkers

## Abstract

Metastatic prostate cancer (PCa) is still an incurable disease. Long non-coding RNAs (lncRNAs) may be an overlooked source of cancer biomarkers and therapeutic targets. We therefore performed RNA sequencing on paired metastatic/non-metastatic PCa xenografts derived from clinical specimens. The most highly up-regulated transcript was LOC728606, a lncRNA now designated PCAT18. PCAT18 is specifically expressed in the prostate compared to 11 other normal tissues (p<0.05) and up-regulated in PCa compared to 15 other neoplasms (p<0.001). Cancer-specific up-regulation of PCAT18 was confirmed on an independent dataset of PCa and benign prostatic hyperplasia samples (p<0.001). PCAT18 was detectable in plasma samples and increased incrementally from healthy individuals to those with localized and metastatic PCa (p<0.01). We identified a PCAT18-associated expression signature (PES), which is highly PCa-specific and activated in metastatic vs. primary PCa samples (p<1E^−4^, odds ratio>2). The PES was significantly associated with androgen receptor (AR) signalling. Accordingly, AR activation dramatically up-regulated PCAT18 expression in vitro and in vivo. PCAT18 silencing significantly (p<0.001) inhibited PCa cell proliferation and triggered caspase 3/7 activation, with no effect on non-neoplastic cells. PCAT18 silencing also inhibited PCa cell migration (p<0.01) and invasion (p<0.01). These results position PCAT18 as a potential therapeutic target and biomarker for metastatic PCa.

## INTRODUCTION

The vast majority of prostate cancer (PCa)-related deaths are attributed to the progression from localized disease to metastatic castration-resistant PCa (mCRPC)[1]. Despite tremendous research efforts, risk stratification of PCa patients at diagnosis is largely dependent on T stage, Gleason grade and plasma PSA levels, a method that overlooks many potentially metastatic cases. Even if aggressive cases are identified earlier, no curative therapeutic options are available for mCRPC [1, 2] and thus there is also a need to identify novel therapeutic targets.

Human transcriptome analysis has recently revealed that most transcribed RNAs are not translated and thus protein-coding genes account for a small percentage of all RNAs [3]. The non-coding transcripts include the well-known ribosomal-, transfer- and micro-RNAs (rRNA, tRNA, miRNA respectively). MiRNA profiling in patient-derived biological fluids is emerging as a powerful tool to differentiate localized and metastatic PCa [4]. Long non-coding RNAs (lncRNAs), transcripts longer than 200bp with no protein-coding function [5], represent a less investigated class of non-coding RNAs. Estimates suggest the number of human lncRNAs rivals that of protein-coding genes, ranging from 10,000 to 20,000 [6]. Despite these large numbers, only a handful of lncRNAs have been characterized. Notably, most characterized lncRNAs display deregulated expression in cancer cells, where they play oncogenic or tumor suppressive functions [6]. A striking feature of some lncRNAs is their tissue-specificity, which prompted some authors to propose them as novel biomarkers and therapeutic targets [6, 7]. Two previously characterized lncRNAs (PCGEM1 and PCA3) are specifically expressed in PCa compared to an array of normal and neoplastic tissues [8, 9]. While the clinical utility of PCGEM1 has yet to be determined, PCA3 is present in urine samples from PCa patients and is able to detect the disease with 77.5% sensitivity and 57.1% specificity [10]. For this reason, a PCA3 test has been approved by the Food and Drug Administration as a diagnostic tool. However, PCA3 levels are not able to discriminate between indolent and clinically aggressive PCa [10]. Functional data on PCA3 and PCGEM1 are still incomplete or are emerging in very recent publications [11-13], so it is not clear that either of them is a viable therapeutic target.

Here, we describe a multi-step strategy to identify lncRNAs associated with PCa progression. First, we profiled lncRNA expression in a pair of non-metastatic/metastatic patient-derived PCa xenografts. The most up-regulated lncRNA was then queried in cancer databases and quantified in samples from localized PCa and mCRPC patients. Through this analysis, we identified a lncRNA (PCAT18) whose expression is: (1) significantly higher in PCa, compared to other benign and neoplastic tissues; (2) detectable in plasma samples and (3) able to discriminate between localized disease and mCRPC. Our functional analyses indicate that PCAT18 is androgen-regulated and a regulator of PCa cell proliferation, invasion, and migration, thereby positioning this gene as a putative therapeutic target.

## RESULTS

### Transcriptomic profiling of paired patient-derived PCa xenografts

**Figure1:**
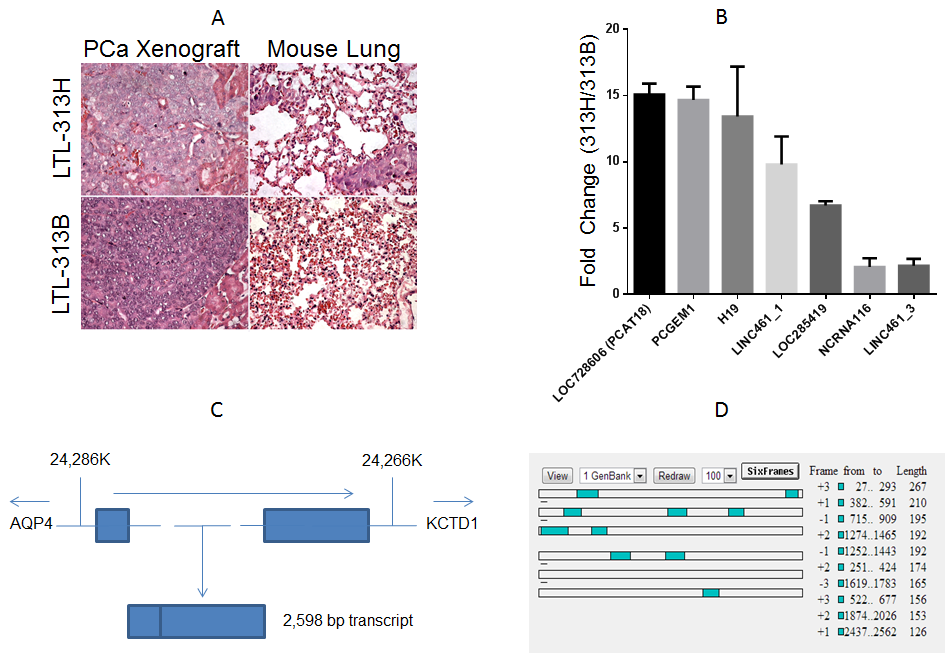
A Hematoxylin-eosin staining of the xenograft (left panels) and mouse lung tissue (right panels) of transplantable prostate cancer tumor lines LTL-313H and LTL-313B. LTL-313H cells are more locally invasive to the adjacent kidney than−313B cells, and show signs of distant metastatic spreading (never found in LTL-313B-engrafted mice). .B, qPCR confirmation of RNA Seq. data (columns represent average value, bars represent standard deviation, 2 replicate experiments). Values indicate relative expression level in LTL-313H *vs.* LTL-313B cells. We chose the 4 most up-regulated and 3 randomly selected transcripts. C, Schematic representation of the PCAT18 *locus* (NLM “Gene” website). The gene is located in a region between 24,286 and 24,266 K (Chromosome 18 primary assembly). Lines represent introns, rectangles represent exons. Dotted lines represent a relative distance that is bigger than the one shown in the picture. Arrows represent transcription direction. The genes flanking PCAT18 *locus* (AQP4, aquaporin-4; KCTD1, Potassium Channel Tetramerization Domain-Containing Protein 1) are shown. D, ORF finder output for PCAT18 sequence. Open Reading Frames are shown as shaded squares throughout the sequence. Each lane represents a possible reading frame. The software identified no ORF longer than 267 bp for a transcript longer than 2Kb. Considering 6 possible reading frames, protein-coding regions could account for no more than 16% of the whole transcript.

The identification of novel biomarkers and therapeutic targets for mCRPC has been hampered by the lack of suitable models that accurately reflect the clinical reality. This hurdle has been overcome by the generation of xenograft models developed from primary patient samples. In the present study we exploited 2 PCa xenograft lines: LTL-313B and LTL-313H (www.livingtumorlab.com). Both models were derived from primary PCa needle biopsies of the same patient, yet they display a strikingly different phenotype. LTL-313B cells showed little local invasion and no distant metastasis within 4 months post-engraftment, while LTL-313H xenografts showed invasion into the mouse host kidney and distant metastases were consistently detectable in the hosts' lungs within 3 months after engraftment (Fig. 1A). Both models show androgen-dependent PSA production and growth.

As the goal of this project was to identify previously uncharacterized lncRNAs associated with PCa metastasis, we performed RNA sequencing analysis on our patient-derived PCa xenografts using the strategy outlined in Suppl. Table. 1. We found 153 up-regulated and 77 down-regulated lncRNAs in metastatic *vs.* non-metastatic xenografts (Suppl. Table 2, 3). The vast majority of these transcripts have not been previously characterized. Of note, the list of up-regulated transcripts included two known oncogenic lncRNAs, H19 and PCGEM1 [9, 14], while the down-regulated transcripts included a well-known onco-suppressive lncRNA in PCa (PTENP1) [15]. PCA3 was detectable in both models, but its differential expression was below the significance threshold (LTL313H vs. LTL313B RPKM ratio=1.38). To validate our RNA sequencing data, we designed primers for 7 differentially modulated lncRNAs and confirmed greater than 2-fold up-regulation for each of them in the metastatic *vs.* non-metastatic xenografts (Fig.1B, primer sequences are listed in Suppl. Table 4).

### Identification of PCAT18, a PCa-specific lncRNA

Among differentially expressed lncRNAs, the transcript with highest expression in the metastatic xenograft was *LOC728606*. This transcript showed a similar magnitude of fold-change with the oncogenic lncRNAs H19 and PCGEM1 (Fig.1B). *LOC728606*, flanked by *AQP4* (Aquaporin-4) and *KCTD1* (Potassium channel tetramerisation domain containing-1) *loci*, encodes for a 2598 bp RNA containing 2 exons (Fig.1C). Transcript length and sequence was confirmed by comparative analysis of multiple clones (Suppl. Table 5). *LOC728606* is classified as a “long intergenic non-coding RNA” based on the Ensembl algorithm (www.ensembl.org). ORF Finder (www.ncbi.nlm.nih.gov/gorf/gorf.html) revealed that the transcript is composed of non-translatable regions for at least 84% of its length (Fig. 1D). Test-code software [16] confirmed that the RNA does not encode a protein (p<0.01), and PepBank [17] failed to identify any human peptide matching any ORF of this *locus*.

**Figure2:**
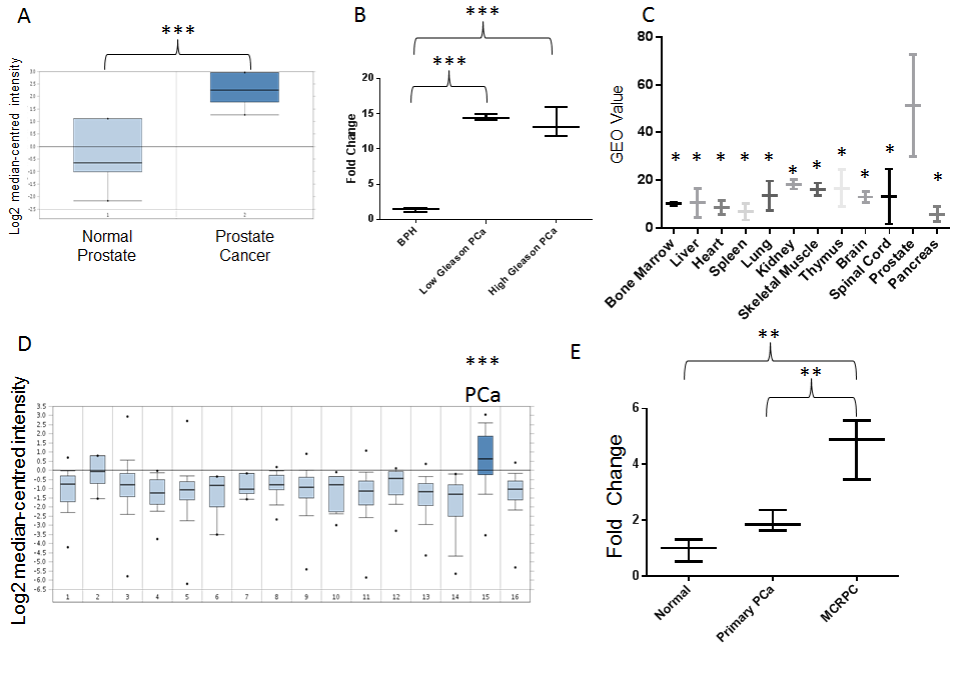
A, PCAT18 expression in normal prostate (n=6) and PCa (n=7) samples (horizontal bar represents median value, vertical bars represent minimum and maximum value per group). Query thresholds for all Oncomine analysis were p<0.01 and fold change>2. All Oncomine outputs passing these thresholds in prostate cancer studies are shown in Fig. 2. The non-significant correlations are summarized in Suppl. Table 6. Oncomine™ database (Compendia Bioscience, Ann Arbor, MI) was used for analysis and visualization. ***p<0.001 (Oncomine Analysis). Fold change: 7.2. B, PCAT18 expression (qPCR) in benign prostatic hyperplasia (BPH, n=5), low-Gleason (n=5) and high-Gleason (n=6) PCa samples. (Median-centered values, bars represent minimum and maximum value per group). ***p<0.001 (ANOVA and Tukey's post-test). C, Expression of PCAT18 in 12 benign tissues (GEO database, http://www.ncbi.nlm.nih.gov/geo/, study ID: HG-U95D), n=2 per tissue, horizontal bar represents mean value, vertical bars represent minimum and maximum value per group. *p<0.05 compared to prostate (ANOVA and Holm-Sidak`s post-test). Fold Change: 2.78-8.75 (prostate compared to other tissues). D, Oncomine analyis of PCAT18 expression in 16 tumor tissues (median-centered values, bars represent minimum and maximum value per group). Data are centered to the median level of expression in the whole cohort. Sample size for each tumor type is in brackets: 1. Bladder Cancer (32); 2. Brain and CNS Cancer (4); 3. Breast Cancer (328); 4. Cervical Cancer (35); 5. Colorectal Cancer (330); 6. Esophageal Cancer (7); 7. Gastric Cancer (7); 8. Head and Neck Cancer (41); 9. Kidney Cancer (254); 10. Liver Cancer (11); 11. Lung Cancer (107); 12. Lymphoma (19); 13. Ovarian Cancer (166); 14. Pancreatic Cancer (19); 15. Prostate Cancer (59); 16. Sarcoma (49). ***p<0.001 E, PCAT18 expression (qPCR) in plasma samples from normal individuals and patients with localized or metastatic castration-resistant (mCRPC) PCa, (median-centered values, bars represent minimum and maximum value per group) **p<0.01 (ANOVA and Tukey's post-test). Samples were processed as previously described [4] for plasma separation, RNA extraction, retrotranscription and quantification. Oncomine™ (Compendia Bioscience, Ann Arbor, MI) was used for analysis and visualization.

To explore the clinical relevance of this gene, we investigated its expression in publically available databases and in human PCa samples. Albeit classical microarray platforms were restricted to mRNA detection, some of them might fortuitously hold probes matching a few lncRNAs [18]. We therefore mined *LOC728606* expression profiles on Oncomine and Gene Expression Omnibus (GEO) databases, which include large collections of microarray data from human samples [19, 20]. Oncomine analysis revealed that *LOC728606* is significantly up-regulated in PCa *vs.* normal tissues (Fig. 2A, Suppl. Table 6). To validate our findings, we measured by qPCR the expression levels of *LOC728606* in human prostate tissues (patients' characteristics are summarized in Suppl. Table 7). This transcript was highly over-expressed (8.8-11.1 fold, p<0.001) in both low-Gleason and high-Gleason PCa samples, compared to benign prostatic hyperplasia (Fig. 2B). This suggests that its up-regulation is not merely the function of prostate cell hyper-proliferation. *LOC728606* expression is significantly higher in normal prostate than in 11 other normal tissues (Fig. 2C). Moreover, this gene is over-expressed in PCa compared to 15 other neoplastic tissues (Fig. 2D). Based on its cancer and tissue-specificity, we sought to determine if *LOC728606* is detectable in plasma samples, and if it can be exploited as a biomarker for disease detection and monitoring, as suggested for other non-coding RNAs [4]. We therefore analyzed plasma samples from normal individuals, and those with localized PCa or mCRPC (n=25 per group, patients' characteristics summarized in Suppl. Table 7). Our results revealed a positive correlation between plasma *LOC728606* levels and disease stage (Fig. 2E, p<0.01 for linear trend test), with significantly higher levels in mCRPC compared to all other categories. To further strengthen our analysis, we confirmed the *LOC728606* expression profile in both pre-clinical and clinical samples, using a new set of primers and a different qPCR methodology (Suppl. Fig. 1 A, B). In light of these data, this gene was officially named PCAT18 (Prostate Cancer-Associated Transcript-18) by the HUGO Gene Nomenclature Committee.

### PCAT18 is an androgen-regulated gene

**Figure 3:**
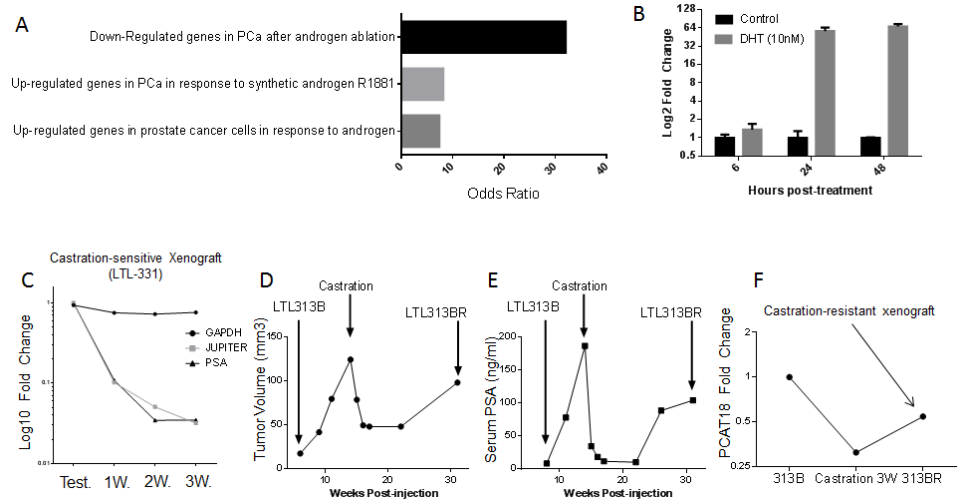
A, Transcripts positively associated with PCAT18 (SAM analysis, Q<0.5%) were analyzed in Oncomine for “literature defined concepts” (p<E^−4^, odds ratio>2).Here we show the top 3 concepts associated with JES. B, PCAT18 expression levels in untreated LNCaP cells (Control) and cells supplemented with dihydrotestosterone (DHT, 10nM, 6-24-48h). LNCaP cells were grown in phenol red-free medium (RPMI-1640) supplemented with 10% charcoal-stripped FBS. Columns represent mean value (2 independent experiments performed in triplicate), bars standard deviation. C, Expression of 3 genes in xenografts from mice supplemented with Testosterone (Test.) (2.5mg/mouse, n=2) or after castration (1, 2, 3 weeks, n=3). LOC728606 (PCAT18) down-regulation is comparable to that of PSA. Data are from LTL-331 human prostate cancer xenografts (www.livingtumorlab.com) and normalized to the average HPRT1 expression level in testosterone-supplemented animals. HPRT1 expression is stable pre- and post-castration (unpublished microarray data). RNA extraction, retro-transcription and QPCR were performed as described in Fig. 1B legend. D, E, The living tumor lab (www.livingtumorlab.com) comprises a collection of patient-derived PCa tumor tissue xenografts, originated with a method described in reference. An androgen-dependent PCa line (LTL313B) has been exposed to castrate-levels of testosterone for a prolonged time, in order to generate a castration-resistant subline. The figures show LTL313B tumor volume (D) and serum PSA levels (E) before and after castration. Neoplastic cells were implanted in male NOD/SCID intact mice, supplemented with testosterone until castration. Serum PSA was measured and mice were sacrificed for tumor volume measurement at indicated time points, as described before [25]. At 12-16 weeks post-castration, a castration-resistant, AR-positive cell line was generated (LTL-313BR). F, PCAT18 expression was measured by qPCR in testosterone-supplemented LTL313B, castrated xenografts (3 weeks) and in a CRPC subline (LTL313BR, no testosterone supplementation).

Based on its expression profile, we hypothesized that PCAT18 might contribute to PCa clinical characteristics and interact with known oncogenic pathways. Therefore, we performed significance analysis of microarray data (SAM) to identify PCAT18-associated transcripts. To this end, we exploited a dataset collecting RNA sequencing data and clinical information on 131 PCa samples and 29 normal prostate tissues [21, 22]. Analysis of this large dataset further confirmed that PCAT18 is significantly (p<0.001) up-regulated in PCa *vs.* normal prostate (data not shown). SAM revealed 402 genes positively and significantly associated with PCAT18 expression (Suppl. Table 8). This PCAT18-associated expression signature (PES) was then uploaded into Oncomine to identify clinically meaningful associations and to perform pathway analysis (thresholds: p<E^−4^ odds ratio>2). PES was consistently up-regulated in PCa *vs.* normal tissue and PCa *vs*. other neoplasms in several cancer studies, comprising more than 4000 human samples (Table 1). More interestingly, PES was significantly activated in metastatic *vs.* primary PCa samples. Pathway analysis revealed that PES is strongly associated with androgen receptor (AR) activation (Fig. 3A).

**Table 1:** PCAT18-associated expression signature (PES) in prostate cancer samples Genes positively associated with PCAT18 (SAM analysis Q<0.5%) were uploaded in the Oncomine databases (thresholds: p<E-4, odds ratio>2). The first column indicates the queried Oncomine concept. The second column (“Studies”) shows the number of independent studies showing up- or down-regulation of PES for a specific concept. Oncomine™ (Compendia Bioscience, Ann Arbor, MI) was used for analysis.

Concept	Studies (Up/Down)	P value	Odds Ratio	Total Samples
PCa vs. Normal	16/0	3.1E-116-1.7E-6	2.4-13.4	928
PCa vs. Other Neoplasms	4/0	3.1E-51-6.9E-7	2.5-6.8	3195
Metastatic vs. Primary PCa	2/0	6.6E-8-2.3 E-5	2.9	27

To gain insights into PCAT18 function, we performed *in vitro* studies on the metastatic PCa-derived LNCaP cell line [23]. In this model, dihydrotestosterone (DHT) treatment (24-48h) induced a more than 50-fold up-regulation of PCAT18 expression (Fig. 3B). In keeping with this relatively late up-regulation, we found no AR binding site in the PCAT18 putative promoter (Suppl. Table 9 and data from ChIP-on-chip[24]). This evidence suggests that AR indirectly activates PCAT18 expression. To experimentally determine whether PCAT18 is down-regulated following androgen ablation, we assessed its expression following castration in two PCa xenograft models. The LTL331 model generates a typical androgen-dependent PCa [25]. In this model, androgen deprivation induced a dramatic PCAT18 down-regulation (Fig. 3C). We then investigated the expression profile of PCAT18 in a recently developed CRPC subline (LTL313BR). When the LTL313B xenograft is exposed to castrate levels of androgens for several weeks, it reproducibly generates an AR+ CRPC subline (Fig. 3D, E) [25]. In this model, castration induced PCAT18 down-regulation, but the emergence of the CRPC subline was associated with PCAT18 up-regulation (Fig. 3F).

### Functional characterization of PCAT18

Our results to this point indicated that PCAT18 is a potential biomarker, is androgen-regulated, and may in turn regulate expression of numerous genes. We next set out to determine the functional relevance of PCAT18 in PCa cells. To this aim, we measured its expression levels in a panel of prostate cell lines. In keeping with our previous data, PCAT18 expression was higher in AR-positive than in AR-negative PCa cells (Suppl. Fig. 1C). Among AR-positive cells, PCAT18 levels incrementally increased from non-neoplastic (BPH1), to androgen-sensitive (22Rv1, LNCaP) and androgen-insensitive (C4-2) PCa cells. We therefore selected LNCaP and its castrate-resistant sub-line C4-2 [26] for lncRNA characterization and functional studies. RNA fractionation and quantification experiments revealed that PCAT18 is mainly located in the cytoplasm of PCa cells (Suppl. Fig. 1 D). Indeed, the PCAT18 expression profile is more similar to the protein-coding RNA GAPDH than to the nuclear-retained lncRNA MALAT1 [27].

**Figure 4:**
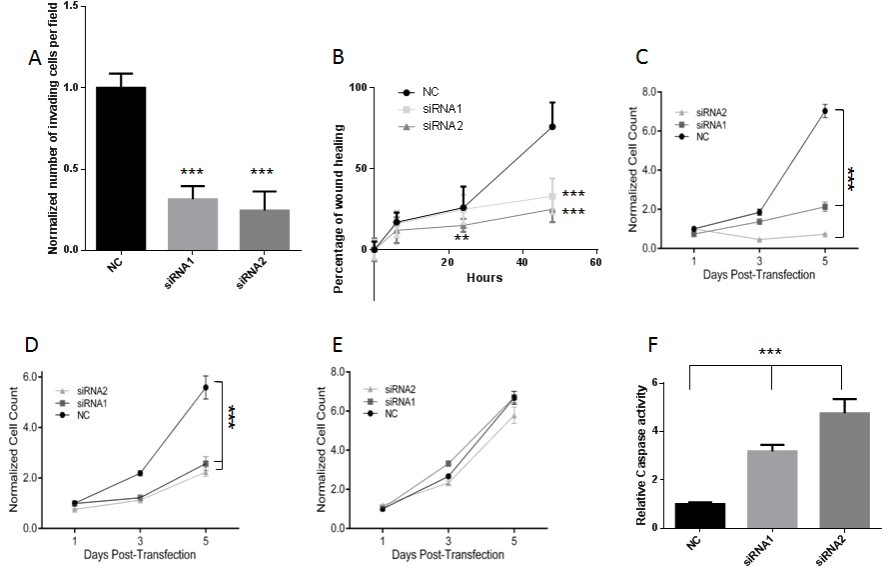
A, C4-2 invasion was quantified 24h after the start of the invasion assay. Cells were transfected with 2nM Negative Control (NC) or PCATT18-targeting siRNA1 and siRNA2. Columns represent mean value (4 experiments) bar SD. ***p<0.001 (ANOVA and Dunnett's post-test). B, C4-2 cell migration was quantified at 6h, 24h or 48h post-transfection, **P<0.01, ***P<0.001 (siRNA *vs.* NC), 2 way ANOVA and Tukey's post-test. C, D, E, MTT assay was performed on LNCaP (C) C4-2 (D) and BPH (E) cells treated with negative control (NC) or PCAT18-targeting siRNAs (both at 2nM concentration) on days 1-3-5 post-transfection, as previously described [30]. Dots represent mean value, lines standard deviation (2 experiments performed in triplicate, data normalized to cell number in NC-day1) ***p<0.001 (2 way ANOVA and Tukey's post-test). F, LNCaP cells were transfected with negative control (NC) or PCAT18-targeting siRNAs for 5 days. Bars represent mean values, lines standard deviations (2 independent experiments performed in triplicate). ***p<0.001 with respect to NC (ANOVA and Dunnet's post-test).

As a further step for PCAT18 characterization, we identified two PCAT18-specific siRNAs inducing greater than 80% gene knockdown at a 2nM concentration (Suppl. Fig. 1E) [28]. PCAT18 silencing (24-48h) significantly inhibited PCa cell invasion and migration (Fig. 4A, B). At later time points (5 days), PCAT18 silencing induced a significant growth inhibition in both LNCaP and C4-2 cells (Fig. 4C, D), with no effect on non-neoplastic BPH1 cells (Fig. 4E). Prolonged PCAT18 silencing (5 days) also triggered caspase 3/7 activation (Fig. 4F).

## DISCUSSION

PCa samples are often composed of multi-clonal subpopulations, each with a different mutational spectrum and metastatic potential [29]. Molecular analysis of PCa samples is therefore affected by this heterogeneity, which often masks the aggressive signature of truly metastatic cells. As a consequence, the development of gene expression profile-based diagnostic and prognostic algorithms is particularly challenging in PCa. To address this concern, we analyzed the transcriptome of tumor tissue lines derived from two primary PCa biopsies of the same patient. When engrafted in the sub-renal capsule of immunocompromised mice, one tumor tissue line invariably generates non-metastatic and relatively non-invasive tumors, while the other line is reproducibly able to generate highly invasive tumors, producing distant metastases through predictable routes. Since the two tumor tissue lines are derived from the same patient they represent the ideal model to study gene expression changes in PCa progression to a metastatic disease. A similar model has been successfully exploited for the identification of PCa-associated miRNAs and protein-coding genes [30, 31].

The heuristic potential of our analysis is confirmed by the characterization of a lncRNA (PCAT18), whose function in human neoplasms had never been described before. Data from 4 independent datasets and more than 600 human samples revealed that this gene is prostate-specific and highly up-regulated in PCa. Even though the PCAT18 transcript has been reported before [32], its function and expression profile has not been previously described. Chinnaiyan and co-workers published a list of unannotated lncRNAs expressed in PCa [33]. Since they deliberately filtered out transcripts present in the RefSeq database they likely excluded PCAT18 from their analysis. More recently, Ren and co-workers published a list of lncRNAs expressed in PCa based on the fRNAdb database [34]. We actively searched for all the sequences matching PCAT18 in this database, finding that this *locus* is covered by just 4 short sequences that span <10% of the entire transcript. Moreover, we were not able to find the PCAT18-matching sequences in Ren's list of PCa-associated lncRNAs. Since Ren *et al.* only analyzed sequences longer than 200 bp. we assume they filtered out the short PCAT18-matching sequences present in the fRNAdb database. Our analysis, which identified putative lncRNAs based on the RefSeq database, thus complements the evidence accumulated by these 2 previous papers, thereby highlighting the importance of multiple analysis methods in the lncRNA field. The expression pattern of PCAT18 resembles that of PCA3 and PCGEM1. Our analysis indicates that PCAT18 is more over-expressed in PCa than PCGEM1 and that a set of patients over-expressing this gene does not express PCA3 (data not shown). Thus our data suggest that PCAT18 can be a valuable addition to a multi-gene platform for use as a non-invasive method for PCa diagnosis. More importantly, since PCAT18 is so frequently over-expressed in PCa cells and is PCa-specific, we speculate that its measurement in plasma samples can allow earlier and more accurate detection of PCa progression to a metastatic and drug-resistant stage. Indeed, identification of PCa-specific nucleotide sequences has been proposed as a tool to improve aggressive PCa detection [4]. Most of those studies were based on the measurement of PCa-associated genomic mutations or microRNAs. In this paper, we showed for the first time that a lncRNA detectable in plasma samples from PCa patients can discriminate between localized and mCRPC.

Consistent with its prostate-specific expression profile, we found that PCAT18 up-regulation is triggered by AR activation (Fig 3). Our promoter analysis indicated that AR is unlikely to directly bind the PCAT18 promoter in keeping with our observations of a relatively late increase in PCAT18 expression following androgen exposure. In PCa cells, AR orchestrates regulatory networks through the activation of several transcription factors [35], some of which have putative binding sites in the PCAT18 promoter (Suppl. Table 9). For example, our RNA Seq. data indicate that c-FOS and PAX5 are highly up-regulated in LTL-313H vs. LTL313B cells (RPKM ratio>7, data not shown). Thus, it is conceivable that AR activates another transcription factor, which in turn is directly responsible for PCAT18 up-regulation. This model is not in contrast with PCAT18's high expression in mCRPC, as PCa progression to a castration-resistant phase still remains dependent on reactivation of this pathway [36]. Indeed, most of the recently approved therapies for mCRPC aim at targeting the AR pathway [1]. Our analyses indicate that PCAT18 is required for invasion, migration and proliferation of castration-resistant PCa cells. The latter effect is probably triggered by the activation of caspase 3 and 7, which orchestrate the demolition phase of apoptosis [37]. Importantly, inhibition of PCAT18 had no effect on non-neoplastic cells. We believe these initial *in vitro* studies pave the way for development of novel therapeutic strategies (*e. g.* antisense oligonucleotides) to verify its function *in vivo* and ultimately be used as PCAT18-targeting drugs for the treatment of metastatic PCa.

## MATERIALS AND METHODS

### Patient-derived xenografts

Primary PCa biopsy specimens were collected at the BC Cancer Agency with the patient's written informed consent. The protocol for this procedure was approved by the University of British Columbia (UBC) Research Ethics Board (REB) (protocol number: H04-60131). NOD/SCID mice were bred and maintained at the British Columbia Cancer Research Centre Animal Facility (Vancouver, Canada). All experimental protocols were approved by the University of British Columbia Animal Care Committee (protocol number: A10-0100). Transplantable PCa tissue xenograft lines were established and maintained using subrenal capsule grafting as previously described [30]. LTL313B and LTL313H tumor tissue cell lines were derived from 2 primary neoplasm biopsies obtained simultaneously from the same patient (total biopsies performed=8). At the time of biopsy, the donor was affected by treatment-naive prostate adenocarcinoma (Gleason Score=8) with signs of pelvic infiltration and bone metastasis. Immediately after pathological diagnosis, the patient received hormonal therapy, and 9 months after commencing treatment PSA reached a nadir 0.28ng/ml from a pre-treatment value of 19 ng/ml.

### RNA Sequencing

Total RNA was extracted from LTL-313B and LTL-313H primary xenografts, harvested on the same day, using Trizol (Invitrogen). RNA was sent to Otogenetics (Norcross, GA) for sequencing. Sequenced reads were aligned to the hg19 human genome assembly and contrasted to the transcriptome generated from all the spliced sequences annotated in the RefSeq database using the DNAnexus suite (www.dnanexus.com). Transcript level was quantified by calculating the RPKM (reads per kilobase of transcript per million mapped reads) value [38]. RPKM values were normalized to the root mean square (RMS) for each sample. Mapped transcripts were annotated using the gene cards database (www.genecards.org). Genes were categorized as “protein coding” and “non-coding” based on their functional annotation. Among non-coding sequences rRNAs, tRNAs, miRNAs snoRNAs and other known classes of RNAs were excluded from further analysis. LncRNAs were defined as all non-coding sequences longer than 200 bp and not belonging to other RNA categories. Based on those filtering criteria, we identified 1653 lncRNAs expressed in PCa xenografts.

### Quantification of lncRNA expression levels

Primers targeting selected lncRNAs were designed using BLAST software. Primer sequences, listed in Suppl. Table 2, were contrasted to the *Homo Sapiens* and *Mus Musculus* trancriptome to ensure their specificity for the intended target gene. Custom DNA oligos were provided by Invitrogen. RNA was extracted by RNAeasy kit (Qiagen) and retro transcribed by QuantiTect kit (Qiagen). The QuantiTect kit includes a genomic DNA elimination step, which was always carefully performed for the experiments presented in this paper. Quantitative PCR was performed as previously described [31] using cDNA, primers and KAPA SYBR fast Universal Master Mix through ABIPrism 7900HT (Applied Biosystems) and following manufacturers' instructions. We used the 2^−ΔΔCT^ method for calculating the fold changes relative to endogenous controls (HPRT and GAPDH, whose expression was stable in primary and metastatic xenografts, according to RNA Seq. data). We were not able to design effective primers for LOC100329109 (a pseudogene). We designed primers specific for the 2 main variants of linc461 (transcript variant 1 LINC461_1 and transcript variant 3 LINC461_3) to confirm separately their up-regulation.

To confirm PCAT18 expression patterns with another methodology, we employed Applied Biyosystem Non-coding RNA assay Hs03669364_m1, which is specific for LOC728606 (PCAT18) and spans the exon1-exon2 boundary. QPCR was performed according to manufacturer`s instructions on the ABIPrism 7900HT (Applied Biosystems). We used the 2^−ΔΔCT^ method for calculating the fold changes relative to endogenous control (GAPDH).

TaqMan qPCR was also performed to quantify the sub-cellular localization of PCAT18. GAPDH and MALAT1 (Hs00273907_s1). Total, cytoplasmic and nuclear RNA was extracted and purified using the Ambion PARIS kit (Life Technologies), following manufacturer`s instruction.

### *In vitro* experiments

Unless otherwise specified, Prostate cancer- and benign prostatic hyperplasia-derived cell lines were maintained in 10% fetal bovine serum (GIBCO, Life Technologies ) and RPMI 1640 growth medium (GIBCO, Life Technologies).

Gene Silencing: cells were treated with 2nM PCAT18 (*LOC728606*)-targeting siRNAs (siRNA1 and siRNA2) or negative control (NC) reagent (Dicer substrate siRNAs, Integrated DNA Technology, Duplex names: NR_024259_1; NR_024259_2; DS_NC1), following manufacturer`s instructions. NC (negative transfection control) is a DsiRNA duplex that does not target any known human or mouse transcript. Lipofectamine RNAiMaX (Invitrogen) was employed as the transfection reagent. RNA extraction, retro-transcription and qPCR were performed as described in Fig. 1B legend.

MTT assay was performed on LNCaP, C4-2 and BPH cells treated with NC or PCAT18-targeting siRNAs (both at 2nM concentration) on days 1-3-5 post-transfection, as previously described [30].

Caspase 3 and 7 activity was quantified through Caspase-Glo 3/7 assay (Promega), as previously described [39] on cells transfected with the above described protocol.

The wound healing assay was performed in triplicate on C4-2 cells as previously described [24]. Transfection protocols were identical to those described above. 12 hours post-transfection, a `wound` was produced using a P20 pipette tip. Pictures were taken at marked spots 0-6-24-48h post-wounding, using a Zeiss Axiovert 40 CFL inverted microscope connected to Axiovision 4.7 software.

Invasion assay was performed in triplicate on C4-2 cells using BD BioCoat™ BD MatrigelTM Invasion Chambers (24-well plates) and following manufacturer's instructions. Transfection was performed on day 0, as described above. After 12 hours, cells were plated in the invasion chambers. 16 hours post-plating, we followed a previously described method for analysis and quantification of invading cells [40].

### Patient datasets

Prostate tissue samples: Samples from patients with benign prostatic hyperplasia (BPH) or PCa were collected at the Stephanshorn Clinic in St. Gallen Switzerland, after study protocol approval by the local ethical committee (protocol number: ZeTuP19/04). Resected specimens were immediately transferred on ice to the Institute for Pathology of the Kantons Hospital, St.Gallen for examination. Small tissue samples from macroscopically visible tumor and non-tumor prostate tissue were dissected, snap frozen in liquid nitrogen and cryo-preserved at –80 °C. Samples were cut in a cryo-microtome and a slide of each probe was stained with hematoxylin-eosin for histological verification. RNA was isolated from frozen materials using the TRI-reagent (Ambion) method according to the manufacturer's guidelines. cDNA was synthesized from 1μg of total RNA using Superscript II RNase H-reverse transcriptase (Invitrogen).

Plasma Samples: Upon study protocol approval by UBC REB (protocol number: H11-00525), and after obtaining written informed consent from study participants, blood samples and clinico-pathological data were collected at the British Columbia Cancer Agency (BCCA), Vancouver Centre. Three cohorts were evaluated: 25 individuals with no clinical sign of neoplasm; 25 PCa patients with treatment-naïve localized disease (Localized PCa); 25 patients with clinically confirmed metastatic PCa and progressive disease despite castration therapy (mCRPC). Risk groups are defined based on pre-prostatectomy serum PSA value, T stage and Gleason Grade, as recommended by Genito-Urinary Radiation Oncologists of Canada [2]. PCa diagnosis was confirmed by pathological examination of tumor biopsies for each enrolled patient. Localized PCa cases were defined as those with no pathological evidence of lymph node dissemination and no clinical evidence of metastatic diffusion. PSA measurement and RNA extraction were performed on samples collected before prostatectomy and on treatment-naive patients. Metastatic cases were defined as those having clinical or pathological evidence of cancer dissemination to any of the following: lymph nodes, bones or soft tissues (lung, brain, spine, testis).

LOC728606 expression was also queried in Oncomine (www.oncomine.com) GEO (www.ncbi.nlm.nih.gov/geo/) and Cbio portal (www.cbioportal.org) gene expression databases. Analysis was restricted to PCa and prostate-derived samples.

Significance Analysis of Microarrays (SAM) was performed in R using the 20 PCa samples expressing the highest and lowest levels of PCAT18 from Cbio database [21] prostate cancer samples [22]. Transcripts positively associated with PCAT18 (with Q<0.5%) were uploaded to the Oncomine database to investigate correlations with clinical variables (threshold: p<E^−4^, odds ratio>2).

### Statistcal analyses

Unless otherwise specified, experiments were repeated at least twice and data were presented and analyzed through GraphPad Prism 6 software.

## SUPPLEMENTARY FIGURE AND TABLES




