# Supplementary material for: Identification of a long non-coding RNA as a novel biomarker and potential therapeutic target for metastatic prostate cancer
Source: Oncotarget. 2014 Feb 6;5(3):764–74. doi: 10.18632/oncotarget.1769 (PMC3996663; doi:10.18632/oncotarget.1769)
Supplement: Supplementary file 1 [file oncotarget-05-764-s001.pdf]

Identification of a long non-coding RNA as a novel biomarker and potential therapeutic target for metastatic prostate cancer – Crea et al

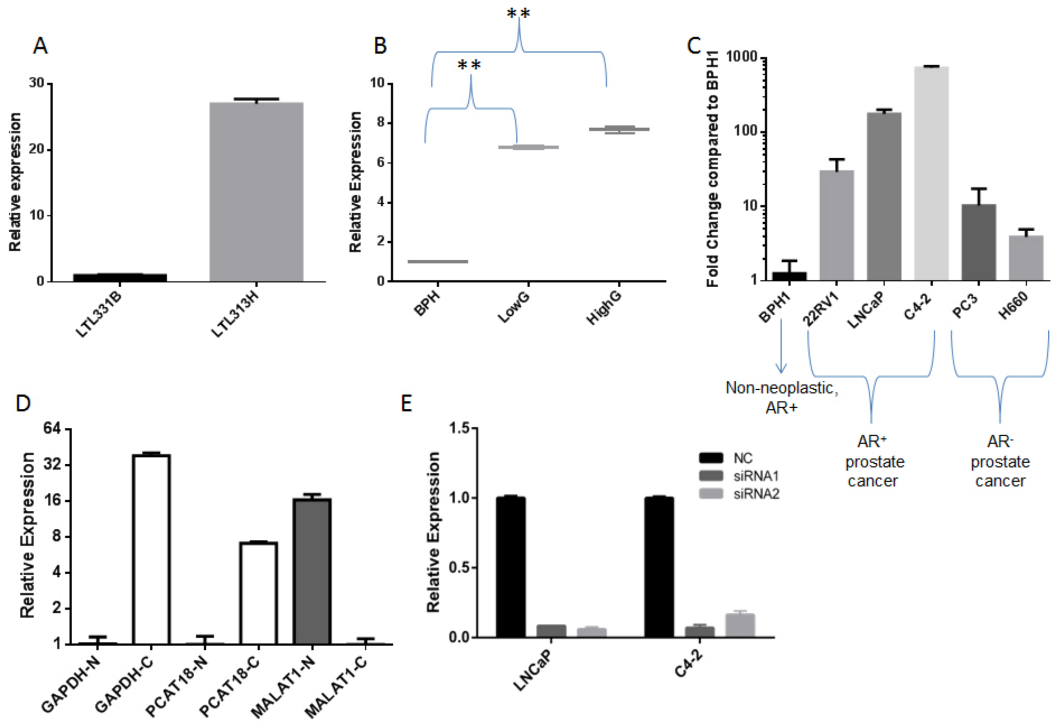

Suppl. Fig. 1:

A, TaqMan qPCR confirmation of PCAT18 expression in PCa xenograft models. Sample type, RNA extraction and retro-transcription methods are identical to those described in Fig 1B.

B, TaqMan qPCR confirmation of PCAT18 expression in clinical samples. Sample type, RNA extraction and retro-transcription methods, as well as statistical analysis are identical to those described in Fig 2B.

C, Basal expression levels of PCAT18 in a panel of prostate cancer cell lines. Columns represent mean values, bars standard deviations (2 independent experiments)

D, Sub-cellular localization of PCAT18, GAPDH and MALAT1. Cellular (C) and Nuclear (N) RNA fractions where extracted and quantified by TaqMan assay, as described in methods. Columns represent mean value, bars standard deviation (2 independent experiments).

E, TaqMan qPCR confirmation of siRNA-mediated PCAT18 silencing (C4-2 cells). Columns represent mean value, bars standard deviation (2 independent experiments).
