# Supplementary material for: Identification of a long non-coding RNA as a novel biomarker and potential therapeutic target for metastatic prostate cancer
Source: Oncotarget. 2014 Feb 6;5(3):764–74. doi: 10.18632/oncotarget.1769 (PMC3996663; doi:10.18632/oncotarget.1769)
Supplement: Supplementary file 2 [file oncotarget-05-764-s002.pdf]

Identification of a long non-coding RNA as a novel biomarker and potential therapeutic target for metastatic prostate cancer – Crea et al

Suppl. Table 1: RNA Seq. analysis on matched non-metastatic (LTL313B) and metastatic (LTL313H) PCa xenografts.

	Non-metastatic	Metastatic
Mapped Reads	17,259,797	15,244,256
Unique Reads	15,711,473	13,704,330
Protein coding genes	18872	18872
Up-regulated lncRNAs (total)	77 (1653)	153 (1653)

Suppl. Table 2: lncRNAs up-regulated in a metastatic (313H) *vs.* localized (313B) PCa xenograft. Displayed genes showed an RMS-normalized RPKM ratio higher than 2 and were ranked based on expression level in 313H cells.

Gene Name	Gene Coordinates	Category	RPKM 313H	RPKM 313B
LOC728606	chr18:24267585-24283602	RNA gene	2.992	0.9714
PCGEM1	chr2:193614571-193641621	RNA gene	2.71	0.3074
LOC100329109	chr2:206980297-206981296	pseudogene	1.242	0.3546
Linc461	chr5:87960264-87969146	RNA gene	0.8182	0.1868
C21orf82	chr21:35552978-35562220	RNA gene	0.7301	0.1746
FLJ40852	chr7:141404138-141438030	RNA gene	0.7177	0.2155
C6orf41	chr6:26924772-26991752	RNA gene	0.6849	0.2271
NCRNA00235	chr16:576847-577407	RNA gene	0.6809	0.237
PMS2L4	chr7:66757424-66767406	pseudogene	0.665	0.2341
NCRNA00092	chr9:98782016-98784037	RNA gene	0.6636	0.2569
FLJ12825	chr12:54452038-54516018	RNA gene	0.6541	0.2586
LOC286094	chr8:136246374-136311959	RNA gene	0.5404	0.1792
PRO0628	chr20:39666473-39667632	uncategorized	0.5351	0.1911
LOC441046	chr4:144480625-144482612	pseudogene	0.5284	0.2453
RPL13AP3	chr14:56232963-56234434	pseudogene	0.519	0.1806
LOC100009676	chr3:101395274-101398055	RNA gene	0.5134	0.1111

LOC644145	chr4:56686237-56703430	pseudogene	0.4923	0.1142
C2orf58	chr2:38358247-38408991	RNA gene	0.4909	0.2278
GTF2IRD2P1	chr7:72656902-72685658	pseudogene	0.4833	0.01473
ISCA1P1	chr5:62071202-62073170	pseudogene	0.4832	0.189
LOC285419	chr4:124695419-124786732	RNA gene	0.4758	0.1405
NCRNA00116	chr2:110969106-110980517	RNA gene	0.4719	0.1095
ADAM6	chr14:106435819-106438358	pseudogene	0.4666	0.1925
TRIM78P	chr11:5664412-5687608	pseudogene	0.4537	0.07019
TYRO3P	chr15:76551630-76552493	pseudogene	0.4421	0.0513
LOC100131193	chr9:139698379-139703300	RNA gene	0.4403	0.1486
RPS10P7	chr1:201489032-201489718	pseudogene	0.4169	0.06443
LOC339568	chr20:37842424-37853391	RNA gene	0.41	0.1464
HAS2AS	chr8:122651586-122656933	RNA gene	0.4079	0.05409
C16orf67	chr16:31711934-31718743	RNA gene	0.4037	0.09994
LOC440839	chr2:113917421-114205429	pseudogene	0.4014	0.187
LOC154761	chr7:143509061-143533810	RNA gene	0.3968	0.1929
HULC	chr6:8652442-8654077	RNA gene	0.3963	0.09196
SNHG12	chr1:28905052-28908366	RNA gene	0.3887	0.1804
C2orf52	chr2:232373137-232379050	RNA gene	0.3725	0.1694
H19	chr11:2016406-2019065	RNA gene	0.3724	0.03841
UOX	chr1:84830648-84863576	pseudogene	0.3711	0.1722

DIRC3	chr2:218148748-218621316	RNA gene	0.3669	0.1476
PCNAP1	chr4:100081753-100082804	pseudogene	0.3631	0.1264
C1orf220	chr1:178511931-178518024	RNA gene	0.3515	0.086
HTR7P1	chr12:13153376-13157762	pseudogene	0.3478	0.1716
CTSLL2	chr10:48155943-48158691	pseudogene	0.3457	0.1203
C6orf176	chr6:166337536-166401527	RNA gene	0.3402	0.07894
LOC1518	chr9:90459660-90462338	pseudogene	0.3363	0.1561
PIPSL	chr10:95717897-95721672	pseudogene	0.3274	0.1408
FLJ41941	chr22:18512151-18520734	RNA gene	0.3211	0.1489
C21orf71	chr21:26955088-26955536	RNA gene	0.319	0.09872
C8ORFK29	chr8:145576887-145578505	RNA gene	0.3159	0.04887
LOC339535	chr1:238643686-238649317	RNA gene	0.3123	0.02899
C21orf41	chr21:30968360-31003067	RNA gene	0.3115	0.08262
MCM3APAS	chr21:47649158-47671604	RNA gene	0.3095	0.1149
LOC257358	chr5:169758435-169762104	RNA gene	0.3033	0.06033
C1orf180	chr1:85093913-85100703	RNA gene	0.298	0.09222
LOC646813	chr11:50368318-50379802	pseudogene	0.298	0.1037
CIDECP	chr3:10059237-10067820	pseudogene	0.2973	0.1104
ZSCAN12P1	chr6:28058929-28063492	pseudogene	0.296	0.1314
LOC389634	chr12:8509562-8543348	RNA gene	0.2898	0.1345
C17orf91	chr17:1614799-1619566	RNA gene	0.2845	0.1337

LOC282997	chr10:112628648-112630662	RNA gene	0.2844	0.06599
LOC146880	chr17:62774258-62777622	RNA gene	0.2837	0.07714
RBMY2FP	chrY:24455006-24462350	pseudogene	0.2809	0.04346
LOC152024	chr3:24141465-24144738	RNA gene	0.277	0.07911
C3orf51	chr3:55691245-55693497	RNA gene	0.2755	0.01967
C14orf132	chr14:96505662-96560133	RNA gene	0.2742	0.1238
LOC100129396	chr17:16692057-16693815	RNA gene	0.2713	0.1008
NCRNA00093	chr10:101686966-101718755	RNA gene	0.2701	0.1337
LOC401010	chr2:132199734-132202467	pseudogene	0.262	0.0486
LOC285692	chr5:9641428-9903936	RNA gene	0.2589	0.02404
LOC100128842	chr1:1193438-1196954	RNA gene	0.2574	0.1008
RPSAP52	chr12:66151803-66220754	pseudogene	0.2511	0.03885
DUSP5P	chr1:228780657-228788159	pseudogene	0.2511	0.07172
FASAS	chr10:90751183-90752732	RNA gene	0.2465	0.1144
PIN1L	chr1:70385005-70386000	pseudogene	0.2397	0.08901
SUMO1P3	chr1:160287055-160288258	pseudogene	0.2379	0.07363
TTC3L	chrX:74960373-74962914	pseudogene	0.2254	0.06971
NCRNA00110	chr21:31120494-31136323	RNA gene	0.2188	0.04063
C14orf34	chr14:56247854-56263392	RNA gene	0.2142	0.06629
NCRNA00052	chr15:88120160-88122917	RNA gene	0.2139	0.04964
SOX2OT	chr3:181328151-181459003	RNA gene	0.2107	0.07112

FLJ14107	chr8:22497884-22499722	RNA gene	0.2077	0.07231
FLJ13224	chr12:31477250-31478879	RNA gene	0.2051	0.02719
NCRNA00174	chr7:65841032-65865395	RNA gene	0.2051	0.08016
C6orf217	chr6:135818939-136011975	RNA gene	0.2044	0.08133
LOC100130522	chr18:77905807-77929616	RNA gene	0.1957	0.05318
LOC400752	chr1:45769582-45771290	RNA gene	0.1956	0.05187
FAM92A3	chr4:183958818-183961271	pseudogene	0.1946	0.05419
C3orf65	chr3:185431040-185435955	RNA gene	0.1945	0.04514
TMPRSS8P	chr16:2889575-2892752	pseudogene	0.1935	0.04489
BTN2A3	chr6:26421619-26430816	pseudogene	0.1923	0.08926
LOC100129534	chr1:2281853-2284100	pseudogene	0.1912	0.07887
LOC255512	chr11:1330938-1331936	RNA gene	0.1912	0.08874
EP400NL	chr12:132568828-132610885	pseudogene	0.1862	0.0576
AKR7L	chr1:19592476-19600568	pseudogene	0.1804	0.06281
BPESC1	chr3:138823027-138844003	RNA gene	0.1768	0.07573
SIGLEC16	chr19:50472912-50479075	pseudogene	0.1763	0.05513
NCRNA00176	chr20:62665697-62671314	RNA gene	0.1734	0.05082
LOC100130015	chr16:90106171-90114033	pseudogene	0.1729	0.06581
TTTY11	chrY:8651359-8685423	RNA gene	0.1728	0.05347
MGC23270	chr14:105287538-105290055	RNA gene	0.1707	0.07041
C9orf106	chr9:132083295-132084882	RNA gene	0.1699	0.05258

C11orf64	chr11:60383224-60454619	RNA gene	0.1687	0.05221
GSTM2P1	chr6:111367623-111368757	pseudogene	0.1683	0.03905
NCRNA00203	chr14:93533797-93538476	RNA gene	0.1632	0.07577
NCRNA00226	chr14:106744269-106744965	RNA gene	0.1608	0.07462
FAM153C	chr5:177435689-177474656	pseudogene	0.1585	0.0735
LOC723809	chr7:104535075-104567092	RNA gene	0.1564	0.07258
LOC653113	chr12:8383646-8395542	pseudogene	0.1548	0.06203
MGC16275	chr17:72206141-72209460	RNA gene	0.1541	0.05364
PRNT	chr20:4711929-4721314	RNA gene	0.153	0.06347
SH3GL1P1	chr17:30367355-30369851	pseudogene	0.1529	0.01773
C4orf12	chr4:85887971-85928168	RNA gene	0.1522	0.0565
LOC285456	chr4:109459346-109541613	RNA gene	0.1519	0.04699
BTF3L1	chr13:77502585-77503223	pseudogene	0.1495	0.06916
INTS4L1	chr7:64601603-64694599	pseudogene	0.1494	0.06938
FLJ40504	chr17:26603012-26634408	pseudogene	0.1469	0.02273
CTSL3	chr9:90387830-90401799	pseudogene	0.1454	0.06747
TUBB4Q	chr4:190903679-190906024	pseudogene	0.1453	0.03393
CYP2D7P1	chr22:42536216-42540575	pseudogene	0.1422	0.05279
C17orf44	chr17:8123949-8127361	RNA gene	0.1369	0.06353
MORF4	chr4:174537088-174537794	pseudogene	0.1351	0.06269
PYY2	chr17:26553589-26555083	pseudogene	0.1341	0.0415

FLJ39534	chr3:47205860-47285605	RNA gene	0.1316	0.05237
LOC650368	chr11:3402191-3430378	pseudogene	0.1293	0.03001
LOC100133957	chrX:47518252-47519776	RNA gene	0.1252	0.05813
HLA-DPB2	chr6:33080293-33096890	pseudogene	0.1186	0.05506
C21orf15	chr21:15215455-15220685	RNA gene	0.1119	0.03464
PPP1R2P9	chrX:42636619-42637486	pseudogene	0.11	0.05107
FLJ40292	chr9:140657474-140659222	RNA gene	0.1092	0.02534
CSDAP1	chr16:31579088-31580845	pseudogene	0.1086	0.05043
LOC643387	chr2:239140327-239142983	pseudogene	0.1078	0.01668
LOC642846	chr12:9436253-9466684	RNA gene	0.1075	0.02531
TDH	chr8:11197146-11225961	pseudogene	0.1056	0.03266
NCRNA00087	chrX:134229015-134232733	RNA gene	0.102	0.04731
PI4KAP1	chr22:20383731-20398695	pseudogene	0.1012	0.03756
UBE2MP1	chr16:34403802-34404762	pseudogene	0.09938	0.04612
SSX8	chrX:52651985-52662998	pseudogene	0.09907	0.03065
NF1P1	chr15:21122021-21134625	pseudogene	0.09875	0.04582
C21orf90	chr21:45937098-45938859	RNA gene	0.09845	0.0457
LOC553137	chr6:107218007-107222877	uncategorized	0.09721	0.0361
LOC154822	chr7:158801045-158818928	RNA gene	0.09521	0.04395
C8orf51	chr8:144448794-144450805	RNA gene	0.09493	0.02203
LOC285501	chr4:178649911-178911903	RNA gene	0.09245	0.04291

TBC1D3P2	chr17:60342069-60353016	pseudogene	0.09224	0.04303
DKFZp434L192	chr7:56563916-56564977	RNA gene	0.08992	0.04174
LOC100189589	chr2:74612845-74621008	RNA gene	0.08909	0.04135
FLJ36000	chr17:21904062-21913070	RNA gene	0.08801	0.03501
FAM35B2	chr10:47379720-47421236	pseudogene	0.08689	0.04029
LOC283761	chr15:90048162-90067265	RNA gene	0.0808	0.03771
LOC149837	chr20:5479218-5485242	RNA gene	0.07978	0.02469
LOC100134259	chr2:47055003-47086145	RNA gene	0.07893	0.03663
LOC100288778	chr12:87984-91262	pseudogene	0.07816	0.03624
C14orf48	chr14:94463642-94478040	RNA gene	0.0749	0.02369
KIAA0125	chr14:106383838-106398500	RNA gene	0.07452	0.01845

Suppl. Table 3: lncRNAs down-regulated in a metastatic (313H) *vs.* localized (313B) PCa xenograft. Displayed genes showed an RMS-normalized RPKM ratio lower than 2 and were ranked based on expression level in 313B cells

Gene Name	Gene Coordinates	Category	RPKM 313H	RPKM 313B
BCL8	chr15:20874797-20961480	pseudogene	1.48	4.125
RPL23AP32	chr2:54756359-54756978	pseudogene	1.494	3.507
GNRHR2	chr1:145509820-145516076	pseudogene	0.9937	3.053
LOC643837	chr1:763064-789740	RNA gene	0.4947	1.406
RPL29P2	chr17:7657638-7658284	pseudogene	0.2952	0.8221
C8orf56	chr8:104145192-104153570	RNA gene	0.2894	0.8059
LOC146481	chr16:34711785-34714967	pseudogene	0.2624	0.6819
LOC286467	chrX:130836679-130964671	RNA gene	0.1293	0.6511
PAR1	chr15:25380789-25383200	RNA gene	0.2574	0.6432
OR4N3P	chr15:22413462-22414393	pseudogene	0.1025	0.6182
BDNFOS	chr11:27528399-27699348	RNA gene	0.2591	0.6119
HPVC1	chr7:54268917-54270114	RNA gene	0.1993	0.592
DLEU2L	chr1:64014651-64016307	pseudogene	0.2305	0.5885
LOC100130987	chr11:67085310-67159158	RNA gene	0.1685	0.5736
SAA3P	chr11:18134020-18137679	pseudogene	0.149	0.5532
SBDSP1	chr7:72299952-72307976	pseudogene	0.1685	0.4984
LOC153684	chr5:43042236-43045370	RNA gene	0.2113	0.4903
LOC440944	chr3:9430537-9439174	RNA gene	0.154	0.4766
RPL13AP6	chr10:112696361-112697013	pseudogene	0.1462	0.4752
LOC284900	chr22:28315364-28398665	RNA gene	0.1402	0.4212

LOC150568	chr2:105050805-105129214	RNA gene	0.1587	0.4126
LOC202781	chr7:154795143-154797412	RNA gene	0.08414	0.41
RPL32P3	chr3:129101678-129118282	pseudogene	0.1151	0.4058
LOC121838	chr13:44596471-44604598	RNA gene	0.1304	0.3632
LOC595101	chr16:30278914-30346695	pseudogene	0.111	0.3606
RAB9P1	chr5:104435175-104435798	pseudogene	0.153	0.3552
LOC100132215	chr2:63271100-63275656	RNA gene	0.05064	0.3525
LOC202181	chr5:177045501-177099278	RNA gene	0.06685	0.3413
LOC100233209	chr12:47602203-47610226	RNA gene	0.1336	0.3366
LOC100101938	chr13:19836941-19919113	pseudogene	0.1282	0.3332
LOC283914	chr16:34597902-34624953	RNA gene	0.1045	0.3233
LOC80054	chr19:33793763-33795962	RNA gene	0.06511	0.3224
SMAD5OS	chr5:135465205-135470579	RNA gene	0.1321	0.3189
LOC648691	chr22:22901756-22909006	RNA gene	0.06153	0.3141
FLJ43390	chr14:62584075-62595131	RNA gene	0.1107	0.3082
C18orf18	chr18:5236724-5238028	RNA gene	0.07318	0.3057
LOC285733	chr6:131148324-131156430	RNA gene	0.1074	0.2992
HEJ1	chr1:102337567-102360299	pseudogene	0.1072	0.2985
LOC285735	chr6:133409219-133427710	RNA gene	0.1187	0.2938
FLJ37307	chr13:52387483-52419286	RNA gene	0.04795	0.2893
CCT6P1	chr7:65216092-65228661	pseudogene	0.1082	0.276
LOC127841	chr1:204337558-204338847	RNA gene	0.111	0.2749
FLJ35390	chr7:44079067-44082081	RNA gene	0.089	0.2685
HSP90B3P	chr1:92100568-	pseudogene	0.07017	0.2605

	92109334			
C1orf213	chr1:23695464-23698278	RNA gene	0.1018	0.2499
LOC284788	chr20:22380971-22401281	RNA gene	0.08953	0.2493
PTENP1	chr9:33673507-33677418	pseudogene	0.08543	0.2492
TMEM191A	chr22:21055402-21058891	RNA gene	0.07316	0.249
MCART3P	chr6:66497772-66499375	pseudogene	0.05954	0.2487
LOC286359	chr9:100153121-100158973	RNA gene	0.09479	0.242
LOC100379224	chr19:44609494-44617336	RNA gene	0.08527	0.2375
LOC728723	chr5:76382623-76444175	RNA gene	0.0837	0.2331
LOC541473	chr7:72440192-72443660	pseudogene	0.09825	0.2279
SYT14L	chr4:68926330-68929015	pseudogene	0.07273	0.2194
GK3P	chr4:166198944-166201175	pseudogene	0.0855	0.2183
ST7OT3	chr7:116822735-116849991	RNA gene	0.09326	0.2164
EGOT	chr3:4790880-4793274	RNA gene	0.06541	0.2125
ARMCX4	chrX:100673266-100788446	RNA gene	0.03532	0.1967
AFG3L1	chr16:90038988-90063028	pseudogene	0.07533	0.1959
SMCR5	chr17:17680000-17682843	RNA gene	0.06716	0.1868
LOC100126784	chr11:19732481-19736146	RNA gene	0.0521	0.1865
PMS2L11	chr7:76610139-76653074	pseudogene	0.05429	0.1763
AMZ2P1	chr17:62962668-62971703	RNA gene	0.02775	0.1674
MGC2889	chr3:192959568-192961760	RNA gene	0.06532	0.1617
LOC339524	chr1:87595448-87602350	RNA gene	0.06287	0.1582
C3orf66	chr3:108897012-108904107	RNA gene	0.05678	0.1581
HCG27	chr6:31165537-31171744	RNA gene	0.05556	0.1547
RRN3P2	chr16:29086163-	pseudogene	0.03989	0.1481

	29128036			
C6orf122	chr6:170188886-170198921	RNA gene	0.0395	0.1466
CFLP1	chr10:89578070-89605365	pseudogene	0.04513	0.1466
LOC647946	chr18:36786888-37331959	RNA gene	0.04448	0.1445
LOC100271722	chr22:46435789-46440748	RNA gene	0.05939	0.1378
HOTAIR	chr12:54356098-54362515	RNA gene	0.04097	0.1141
LOC619207	chr10:135267432-135281949	pseudogene	0.03898	0.09046
ASFMR1	chrX:146990949-147003676	RNA gene	0.03246	0.08789
ALOX12P2	chr17:6756895-6803667	pseudogene	0.03451	0.0801
LOC100303728	chrX:118599997-118603083	RNA gene	0.03094	0.07179

Suppl. Table 4: Primers used for qPCR.

Gene	Forward Primer	Reverse Primer
HPRT1	GGTCAGGCAGTATAATCCAAAG	CGATGTCAATAGGACTCCAGATG
GAPDH	CACCAGGGCTGCTTTTAACTC	GACAAGCTTCCCGTTCTCAG
PCAT18	AGGAGACAGGCCCCAGATTT	TGAAGTGCTGGGACAACGTA
PCGEM1	TTGCCCTATGCCGTAACCTG	ACGTTGAGTCCCAGTGCATC
H19	CCAGTGAGGAGTGTGGAGTAG	CAGCTGCCACGTCCTGTAAC
Linc461_1	AGGAAACAGCTCTGGCATCC	CAGATTCCCCACCCCCTTTC
Linc461_3	GTTCCTGCCCAGCTGGATTT	TCAGAGTAGTCCACGCCAGA
LOC285419	TGACTCAACTTCTGGTGCAGAT	GGATGTGGCATATCTCTTGGTTTA
NCRNA116	GAGACTGCTCAGAGGAAGAGAA	CAGACAGCCCAGTGTCTTGG
KLK3 (PSA)	AGTGCGAGAAGCATTCCCAAC	CCAGCAAGATCACGCTTTTGTT

Suppl. Table 5. Summary of all sequenced clones from *LOC728606 (PCAT18)*. All known clones matching the *LOC728606* sequence (NR_024259.1) were searched through the AceView database. We found 8 sequences, 5 of which were from prostate tissue and 3 from neoplastic tissues. The 5' end of the gene is confirmed by independent readings. The AK056805 clone was generated using a 5' oligo-capping method and polydT primers (described in Ref. 30). The reference sequence was derived from AK056805.1 and DA865211. Match mRNA is antisense strand (AS) for all reads. We analyzed the polyA signal of the NP_024259.1 and AK056805.1 clones. A PolyA signal, AATAAA, was identified -25 to -18 bases from the 3'-end.

cDNA accession	Tissue	Match mRNA	From bp to bp in mRNA	From bp to bp in accs.	Accession match over (% length)	Base differences relative to genome (% identity)
AK056805	Prostate	AS	1 to 2597	2 to 2598	2598/2598 (100 %)	9 diff (99.7 %id)
DA865211	Prostate	AS	1 to 824	2 to 826	825/825 (100 %)	12 diff (98.6 %id)
BX119491	poorly differentiated adenocarcinoma , stomach	AS	245 to 688	458 to 15	444/444 (100 %)	0 diff (100 %id)
AI685598	Prostate	AS	245 to 687	452 to 10	443/468 (94 %)	0 diff (100 %id)
FN152668	breast carcinoma	AS	358 to 447	1 to 90	90/90 (100 %)	0 diff (100 %id)
AI926047	poorly differentiated adenocarcinoma with signet ring cell features, stomach	AS	409 to 690	290 to 8	283/290 (97 %)	0 diff (100 %id)
AA635604	Prostate	AS	428 to 691	264 to 1	264/264 (100 %)	0 diff (100 %id)
DB329187	Prostate	AS	2162 to 2597	439 to 1	439/444 (98 %)	0 diff (100 %id)

Suppl. Table 6: Summary of all Oncomine outputs for *LOC728606* in PCa, with p value >0.01 and/or fold change<2. We selected all PCa studies with patient data included in the Oncomine database.

Study	Comparison	P value	Fold Change	Samples
Arredouani	PCa vs. normal prostate	0.043	2.0	21
Arredouani	ERG rearrangement vs. no rearrangment	0.075	2.0	13
Bittner	PCa-smoker vs. non-smoker	0.503	-1.0	46
Bittner	Acinar PCa-Grade 2 vs. grade 3	0.750	-1.80	10
Bittner	PCa-Grade 2/3 vs. grade 3	0.761	N.A.	46
Bittner	PCa-Stage 2/3 vs. stage 4	0.750	N.A.	43
Bittner	Acinar PCa-smoker vs. non-smoker	0.903	-2.2	11

Table 7: Clinical- pathological characteristics of enrolled patients.

PROSTATE TISSUE SAMPLES (N=16)		
	Prostate cancer	Benign Prostatic Hyperplasia
Median Age (Range)	63 (58-75)	62 (52-67)
Median PSA at diagnosis (Range)	8 (4.5-23.1) ng/ml	4.1 (2.9-5.3) ng/ml
Gleason Score		
≤ 8	5	N.A.
≥ 8	6	N.A.
TNM Stage		
T2a /N0/M0	2	N.A.
T2c /N0/M0	5	N.A.
T3/N0/M0	4	N.A.

PLASMA SAMPLES FROM PCA PATIENTS (N=50)		
	Localized Prostate Cancer	mCRPC
Median Age (Range)	67 (51-82)	73 (45-86)
Median PSA at diagnosis (Range)	9.2 (0.6-22) ng/ml	120 (6.3-4948.5) ng/ml
Risk Group		
Intermediate	22	N.A.
Low	3	N.A.
High		
Metastatic Sites		
Bone	N.A.	17
Lymph nodes and Others	N.A.	5
Others	N.A.	3

Suppl. Table 8: PCAT18-associated expression signature. Genes that were identified as positively associated with PCAT18 by SAM (Q<0.5%) on PCa clinical samples were uploaded to the Oncomine database for clinico-pathological and molecular pathway analysis.

ACACA	acetyl-CoA carboxylase alpha
ACADL	acyl-CoA dehydrogenase, long chain
ACN9	ACN9 homolog (S. cerevisiae)
ACP6	acid phosphatase 6, lysophosphatidic
ACSM1	acyl-CoA synthetase medium-chain family member 1
ACSM3	acyl-CoA synthetase medium-chain family member 3
ACSS1	acyl-CoA synthetase short-chain family member 1
ACY1	aminoacylase 1
ADARB2	adenosine deaminase, RNA-specific, B2 (RED2 homolog rat)
ADRB1	adrenergic, beta-1-, receptor
ADRB2	adrenergic, beta-2-, receptor, surface
AGA	aspartylglucosaminidase
AGAP1	ArfGAP with GTPase domain, ankyrin repeat and PH domain 1
AGFG2	ArfGAP with FG repeats 2
AK2P2	adenylate kinase 2 pseudogene 2
ALDH1A3	aldehyde dehydrogenase 1 family, member A3
ALG14	asparagine-linked glycosylation 14 homolog (S. cerevisiae)
ALKBH2	alkB, alkylation repair homolog 2 (E. coli)
ALMS1P	Alstrom syndrome 1 pseudogene
ANAPC5	anaphase promoting complex subunit 5
ANK3	ankyrin 3, node of Ranvier (ankyrin G)
ANKRD37	ankyrin repeat domain 37
ANKRD5	ankyrin repeat domain 5
AP2S1	adaptor-related protein complex 2, sigma 1 subunit
APOF	apolipoprotein F
ARF4P3	ADP-ribosylation factor 4 pseudogene 3
ARHGAP28	Rho GTPase activating protein 28
ATP5G2	ATP synthase, H+ transporting, mitochondrial F0 complex, subunit C2 (subunit 9)
ATP8A1	ATPase, aminophospholipid transporter (APLT), class I, type 8A, member 1
ATPIF1	ATPase inhibitory factor 1
BAIAP3	BAI1-associated protein 3
BCAM	basal cell adhesion molecule (Lutheran blood group)
BEND4	BEN domain containing 4

BIK	BCL2-interacting killer (apoptosis-inducing)
BOLA3	bolA homolog 3 (E. coli)
BPHL	biphenyl hydrolase-like (serine hydrolase)
BTBD11	BTB (POZ) domain containing 11
C10orf75	chromosome 10 open reading frame 75
C11orf10	chromosome 11 open reading frame 10
C11orf75	chromosome 11 open reading frame 75
C12orf60	chromosome 12 open reading frame 60
C14orf149	chromosome 14 open reading frame 149
C15orf23	chromosome 15 open reading frame 23
C15orf33	chromosome 15 open reading frame 33
C15orf61	chromosome 15 open reading frame 61
C16orf13	chromosome 16 open reading frame 13
C16orf70	chromosome 16 open reading frame 70
C17orf61	chromosome 17 open reading frame 61
C17orf79	chromosome 17 open reading frame 79
C18orf22	chromosome 18 open reading frame 22
C19orf46	chromosome 19 open reading frame 46
C19orf48	chromosome 19 open reading frame 48
C1orf66	chromosome 1 open reading frame 66
C2	complement component 2
C20orf196	chromosome 20 open reading frame 196
C20orf3	chromosome 20 open reading frame 3
C20orf96	chromosome 20 open reading frame 96
C22orf32	chromosome 22 open reading frame 32
C2orf72	chromosome 2 open reading frame 72
C2orf76	chromosome 2 open reading frame 76
C2orf79	chromosome 2 open reading frame 79
C3orf25	chromosome 3 open reading frame 25
C4orf14	chromosome 4 open reading frame 14
C4orf47	chromosome 4 open reading frame 47
C5orf49	chromosome 5 open reading frame 49
C6orf108	chromosome 6 open reading frame 108
C6orf124	chromosome 6 open reading frame 124
C6orf57	chromosome 6 open reading frame 57
C7orf53	chromosome 7 open reading frame 53
C8orf34	chromosome 8 open reading frame 34
C8orf45	chromosome 8 open reading frame 45
C9orf152	chromosome 9 open reading frame 152
C9orf43	chromosome 9 open reading frame 43
CAMK1	calcium/calmodulin-dependent protein kinase I
CAMK2B	calcium/calmodulin-dependent protein kinase II beta
CAMKK2	calcium/calmodulin-dependent protein kinase kinase 2, beta

CAPN9	calpain 9
CATSPER2	cation channel, sperm associated 2
CATSPER2P1	cation channel, sperm associated 2 pseudogene 1
CBS	cystathionine-beta-synthase
CCDC110	coiled-coil domain containing 110
CCDC149	coiled-coil domain containing 149
CCDC51	coiled-coil domain containing 51
CCT3	chaperonin containing TCP1, subunit 3 (gamma)
CDK3	cyclin-dependent kinase 3
CDK5	cyclin-dependent kinase 5
CECR5	cat eye syndrome chromosome region, candidate 5
CECR7	cat eye syndrome chromosome region, candidate 7 (non-protein coding)
CGREF1	cell growth regulator with EF-hand domain 1
CHDH	choline dehydrogenase
CHKA	choline kinase alpha
CHMP4C	chromatin modifying protein 4C
CHRNA2	cholinergic receptor, nicotinic, alpha 2 (neuronal)
CISD3	CDGSH iron sulfur domain 3
CLDN8	claudin 8
CLEC18A	C-type lectin domain family 18, member A
CLEC18B	C-type lectin domain family 18, member B
CLEC18C	C-type lectin domain family 18, member C
CMTM4	CKLF-like MARVEL transmembrane domain containing 4
CNTN3	contactin 3 (plasmacytoma associated)
CORO1B	coronin, actin binding protein, 1B
CPNE7	copine VII
CREB3L4	cAMP responsive element binding protein 3-like 4
CRYL1	crystallin, lambda 1
CYB5A	cytochrome b5 type A (microsomal)
DAK	dihydroxyacetone kinase 2 homolog (S. cerevisiae)
DBI	diazepam binding inhibitor (GABA receptor modulator, acyl-CoA binding protein)
DCXR	dicarbonyl/L-xylulose reductase
DECR2	2,4-dienoyl CoA reductase 2, peroxisomal
DKFZP686I152 17	hypothetical LOC401232
DNAH5	dynein, axonemal, heavy chain 5
DNAH7	dynein, axonemal, heavy chain 7
DOPEY2	dopey family member 2
DPY19L2P4	dpy-19-like 2 pseudogene 4 (C. elegans)
DSC2	desmocollin 2
DUS1L	dihydrouridine synthase 1-like (S. cerevisiae)
EDEM3	ER degradation enhancer, mannosidase alpha-like 3
EGF	epidermal growth factor

EIF4EBP1	eukaryotic translation initiation factor 4E binding protein 1
ELL3	elongation factor RNA polymerase II-like 3
ELMO3	engulfment and cell motility 3
ELOVL5	ELOVL family member 5, elongation of long chain fatty acids (FEN1/Elo2, SUR4/Elo3-like, yeast)
ENOX1	ecto-NOX disulfide-thiol exchanger 1
EPB41L4B	erythrocyte membrane protein band 4.1 like 4B
ERBB3	v-erb-b2 erythroblastic leukemia viral oncogene homolog 3 (avian)
ERGIC1	endoplasmic reticulum-golgi intermediate compartment (ERGIC) 1
ESRP2	epithelial splicing regulatory protein 2
EXOSC5	exosome component 5
FAH	fumarylacetoacetate hydrolase (fumarylacetoacetase)
FAM128A	family with sequence similarity 128, member A
FAM13C	family with sequence similarity 13, member C
FAM19A4	family with sequence similarity 19 (chemokine (C-C motif)-like), member A4
FAM81A	family with sequence similarity 81, member A
FASN	fatty acid synthase
FBXL8	F-box and leucine-rich repeat protein 8
FHIT	fragile histidine triad gene
FLJ27352	hypothetical LOC145788
FLJ46552	FLJ46552 protein
FRMPD3	FERM and PDZ domain containing 3
FZD8	frizzled homolog 8 (Drosophila)
GALNT3	UDP-N-acetyl-alpha-D-galactosamine:polypeptide N-acetylgalactosaminyltransferase 3 (GalNAc-T3)
GGCT	gamma-glutamylcyclotransferase
GJB1	gap junction protein, beta 1, 32kDa
GLYATL1	glycine-N-acyltransferase-like 1
GMPPB	GDP-mannose pyrophosphorylase B
GRB14	growth factor receptor-bound protein 14
GRPR	gastrin-releasing peptide receptor
GTF3C1	general transcription factor IIIC, polypeptide 1, alpha 220kDa
H2AFJ	H2A histone family, member J
HEBP2	heme binding protein 2
HIST3H2A	histone cluster 3, H2a
HKR1	HKR1, GLI-Kruppel zinc finger family member
HMG20B	high-mobility group 20B
HOXA9	homeobox A9
HPN	hepsin
HSF4	heat shock transcription factor 4
ICT1	immature colon carcinoma transcript 1
IGSF5	immunoglobulin superfamily, member 5
IGSF8	immunoglobulin superfamily, member 8

IL20RA	interleukin 20 receptor, alpha
ILDR1	immunoglobulin-like domain containing receptor 1
IMPDH2	IMP (inosine 5'-monophosphate) dehydrogenase 2
IQCH	IQ motif containing H
IVD	isovaleryl-CoA dehydrogenase
KATNB1	katanin p80 (WD repeat containing) subunit B 1
KCNH6	potassium voltage-gated channel, subfamily H (eag-related), member 6
KCTD1	potassium channel tetramerisation domain containing 1
KIAA0182	KIAA0182
KIAA1543	KIAA1543
KIAA1549	KIAA1549
KIAA1804	mixed lineage kinase 4
KLK15	kallikrein-related peptidase 15
KRT18	keratin 18
KRT18P13	keratin 18 pseudogene 13
KRT18P17	keratin 18 pseudogene 17
KRT18P19	keratin 18 pseudogene 19
KRT18P24	keratin 18 pseudogene 24
KRT18P26	keratin 18 pseudogene 26
KRT18P28	keratin 18 pseudogene 28
KRT18P30	keratin 18 pseudogene 30
KRT18P33	keratin 18 pseudogene 33
KRT18P34	keratin 18 pseudogene 34
KRT18P40	keratin 18 pseudogene 40
KRT18P42	keratin 18 pseudogene 42
KRT18P46	keratin 18 pseudogene 46
LASS4	LAG1 homolog, ceramide synthase 4
LEKR1	leucine, glutamate and lysine rich 1
LFNG	LFNG O-fucosylpeptide 3-beta-N-acetylglucosaminyltransferase
LOC100127980	hypothetical protein LOC100127980
LOC100128332	hypothetical protein LOC100128332
LOC100128737	hypothetical LOC100128737
LOC100128841	similar to hCG39453
LOC100129387	hypothetical LOC100129387
LOC100129514	hypothetical LOC100129514
LOC100131047	hypothetical protein LOC100131047
LOC100131199	hypothetical LOC100131199
LOC100132111	hypothetical LOC100132111
LOC100133580	hypothetical protein LOC100133580
LOC100134348	similar to TBC1 domain family member 3 (Rab GTPase-activating protein PRC17) (Prostate cancer gene 17 protein) (TRE17 alpha protein)
LOC389768	potassium channel tetramerisation domain containing 1 pseudogene
LOC391811	similar to polymerase (DNA directed), delta 2, regulatory subunit

LOC399815	chromosome 10 open reading frame 88 pseudogene
LOC440335	hypothetical LOC440335
LOC442249	similar to keratin 18
LOC642384	hypothetical LOC642384
LOC642590	similar to spermine synthase
LOC643327	hypothetical LOC643327
LOC643637	similar to hCG1729961
LOC646347	similar to spermine synthase
LOC653380	TBC1 domain family member 3C-like protein ENSP00000341742
LOC728431	hypothetical LOC728431
LOC729774	hypothetical LOC729774
LOC729779	similar to phosphoserine aminotransferase
LOC81691	exonuclease NEF-sp
LRGUK	leucine-rich repeats and guanylate kinase domain containing
LRIG1	leucine-rich repeats and immunoglobulin-like domains 1
LRRC26	leucine rich repeat containing 26
LRRC63	leucine rich repeat containing 63
LRRIQ1	leucine-rich repeats and IQ motif containing 1
LYPLA2P1	lysophospholipase II pseudogene 1
MAP1D	methionine aminopeptidase 1D
MBOAT2	membrane bound O-acyltransferase domain containing 2
MCCC2	methylcrotonoyl-CoA carboxylase 2 (beta)
MDH2	malate dehydrogenase 2, NAD (mitochondrial)
MED12L	mediator complex subunit 12-like
METTL9	methyltransferase like 9
MMP26	matrix metallopeptidase 26
MOSC1	MOCO sulphurase C-terminal domain containing 1
MPND	MPN domain containing
MRPL12	mitochondrial ribosomal protein L12
MRPL24	mitochondrial ribosomal protein L24
MRPS24	mitochondrial ribosomal protein S24
MRPS33	mitochondrial ribosomal protein S33
MYBPC1	myosin binding protein C, slow type
MYRIP	myosin VIIA and Rab interacting protein
NAAA	N-acylethanolamine acid amidase
NDUFA8	NADH dehydrogenase (ubiquinone) 1 alpha subcomplex, 8, 19kDa
NDUFB10	NADH dehydrogenase (ubiquinone) 1 beta subcomplex, 10, 22kDa
NEIL1	nei endonuclease VIII-like 1 (E. coli)
NME4	non-metastatic cells 4, protein expressed in
NMRAL1	NmrA-like family domain containing 1
NPY	neuropeptide Y
NSUN7	NOP2/Sun domain family, member 7
NUDT9	nudix (nucleoside diphosphate linked moiety X)-type motif 9

NUPR1	nuclear protein, transcriptional regulator, 1
NWD1	NACHT and WD repeat domain containing 1
OAZ3	ornithine decarboxylase antizyme 3
OCEL1	occludin/ELL domain containing 1
OR51A7	olfactory receptor, family 51, subfamily A, member 7
OR51F1	olfactory receptor, family 51, subfamily F, member 1
OR51F2	olfactory receptor, family 51, subfamily F, member 2
OR51G2	olfactory receptor, family 51, subfamily G, member 2
OR51L1	olfactory receptor, family 51, subfamily L, member 1
OR51T1	olfactory receptor, family 51, subfamily T, member 1
OVGP1	oviductal glycoprotein 1, 120kDa
OXSM	3-oxoacyl-ACP synthase, mitochondrial
PAOX	polyamine oxidase (exo-N4-amino)
PCA3	prostate cancer antigen 3 (non-protein coding)
PCBD1	pterin-4 alpha-carbinolamine dehydratase/dimerization cofactor of hepatocyte nuclear factor 1 alpha
PCGEM1	prostate-specific transcript 1 (non-protein coding)
PCGF1	polycomb group ring finger 1
PCGF3	polycomb group ring finger 3
PCTP	phosphatidylcholine transfer protein
PDCD2L	programmed cell death 2-like
PDE3B	phosphodiesterase 3B, cGMP-inhibited
PDE9A	phosphodiesterase 9A
PECI	peroxisomal D3,D2-enoyl-CoA isomerase
PET112L	PET112-like (yeast)
PEX10	peroxisomal biogenesis factor 10
PEX7	peroxisomal biogenesis factor 7
PIGM	phosphatidylinositol glycan anchor biosynthesis, class M
PKN1	protein kinase N1
PLCB4	phospholipase C, beta 4
PMM1	phosphomannomutase 1
PODXL2	podocalyxin-like 2
POLD2	polymerase (DNA directed), delta 2, regulatory subunit 50kDa
POLN	polymerase (DNA directed) nu
POP7	processing of precursor 7, ribonuclease P/MRP subunit (S. cerevisiae)
PPAPDC1B	phosphatidic acid phosphatase type 2 domain containing 1B
PPM1E	protein phosphatase, Mg2+/Mn2+ dependent, 1E
PPM1H	protein phosphatase, Mg2+/Mn2+ dependent, 1H
PPP1R9A	protein phosphatase 1, regulatory (inhibitor) subunit 9A
PPYR1	pancreatic polypeptide receptor 1
PRDM10	PR domain containing 10
PRDX4	peroxiredoxin 4
PRSS8	protease, serine, 8

PRTG	protogenin homolog (Gallus gallus)
PSTK	phosphoseryl-tRNA kinase
PTPN20A	protein tyrosine phosphatase, non-receptor type 20A
PTPN20B	protein tyrosine phosphatase, non-receptor type 20B
PTPRN2	protein tyrosine phosphatase, receptor type, N polypeptide 2
PYCR1	pyrroline-5-carboxylate reductase 1
RAB17	RAB17, member RAS oncogene family
RAB3B	RAB3B, member RAS oncogene family
RAB3D	RAB3D, member RAS oncogene family
RAB3IP	RAB3A interacting protein (rabin3)
RABIF	RAB interacting factor
RAC3	ras-related C3 botulinum toxin substrate 3 (rho family, small GTP binding protein Rac3)
REPS2	RALBP1 associated Eps domain containing 2
RG9MTD2	RNA (guanine-9-) methyltransferase domain containing 2
RIMKLA	ribosomal modification protein rimK-like family member A
RNLS	renalase, FAD-dependent amine oxidase
RORC	RAR-related orphan receptor C
RPL14P3	ribosomal protein L14 pseudogene 3
RPL22L1	ribosomal protein L22-like 1
RPL29P15	ribosomal protein L29 pseudogene 15
RPL29P30	ribosomal protein L29 pseudogene 30
RPL36	ribosomal protein L36
RPL7AP68	ribosomal protein L7a pseudogene 68
RPLP2P3	ribosomal protein, large, P2 pseudogene 3
RPS12P23	ribosomal protein S12 pseudogene 23
RPS19BP1	ribosomal protein S19 binding protein 1
RPS24	ribosomal protein S24
SATB2	SATB homeobox 2
SCAND3	SCAN domain containing 3
SCD	stearoyl-CoA desaturase (delta-9-desaturase)
SHANK2	SH3 and multiple ankyrin repeat domains 2
SLC19A1	solute carrier family 19 (folate transporter), member 1
SLC25A33	solute carrier family 25, member 33
SLC25A42	solute carrier family 25, member 42
SLC26A6	solute carrier family 26, member 6
SLC35F2	solute carrier family 35, member F2
SLC43A1	solute carrier family 43, member 1
SLC9A2	solute carrier family 9 (sodium/hydrogen exchanger), member 2
SMOX	spermine oxidase
SMPDL3B	sphingomyelin phosphodiesterase, acid-like 3B
SMS	spermine synthase
SNHG11	small nucleolar RNA host gene 11 (non-protein coding)

SNORA18	small nucleolar RNA, H/ACA box 18
SNORA2A	small nucleolar RNA, H/ACA box 2A
SNORD104	small nucleolar RNA, C/D box 104
SNORD116-11	small nucleolar RNA, C/D box 116-11
SNORD35A	small nucleolar RNA, C/D box 35A
SNORD57	small nucleolar RNA, C/D box 57
SNORD74	small nucleolar RNA, C/D box 74
SNRPD2	small nuclear ribonucleoprotein D2 polypeptide 16.5kDa
SPAG6	sperm associated antigen 6
SPATA17	spermatogenesis associated 17
SPDEF	SAM pointed domain containing ets transcription factor
SPIN3	spindlin family, member 3
SPOCK1	sparc/osteonectin, cwcv and kazal-like domains proteoglycan (testican) 1
SREBF1	sterol regulatory element binding transcription factor 1
STX19	syntaxin 19
STYXL1	serine/threonine/tyrosine interacting-like 1
TAAR6	trace amine associated receptor 6
TARS2	threonyl-tRNA synthetase 2, mitochondrial (putative)
TAS2R10	taste receptor, type 2, member 10
TBC1D3	TBC1 domain family, member 3
TBC1D3B	TBC1 domain family, member 3B
TBC1D3C	TBC1 domain family, member 3C
TBC1D3E	TBC1 domain family, member 3E
TBC1D3F	TBC1 domain family, member 3F
TBC1D3G	TBC1 domain family, member 3G
TBC1D3H	TBC1 domain family, member 3H
TBC1D4	TBC1 domain family, member 4
TDRKH	tudor and KH domain containing
TERC	telomerase RNA component
TGM3	transglutaminase 3 (E polypeptide, protein-glutamine-gamma-glutamyltransferase)
TMED3	transmembrane emp24 protein transport domain containing 3
TMEFF2	transmembrane protein with EGF-like and two follistatin-like domains 2
TMEM144	transmembrane protein 144
TMEM223	transmembrane protein 223
TMEM27	transmembrane protein 27
TMEM5	transmembrane protein 5
TMPRSS11F	transmembrane protease, serine 11F
TMSB15A	thymosin beta 15a
TMTC4	transmembrane and tetratricopeptide repeat containing 4
TOM1L1	target of myb1 (chicken)-like 1
TP53TG1	TP53 target 1 (non-protein coding)
TREX1	three prime repair exonuclease 1
TRIM3	tripartite motif-containing 3

TSPAN1	tetraspanin 1
TSSC1	tumor suppressing subtransferable candidate 1
TTC18	tetratricopeptide repeat domain 18
TTC6	tetratricopeptide repeat domain 6
TTLL12	tubulin tyrosine ligase-like family, member 12
TUBA3D	tubulin, alpha 3d
TUBA3E	tubulin, alpha 3e
TUT1	terminal uridylyl transferase 1, U6 snRNA-specific
UAP1	UDP-N-acteylglucosamine pyrophosphorylase 1
UBE2E2	ubiquitin-conjugating enzyme E2E 2 (UBC4/5 homolog, yeast)
UBXN8	UBX domain protein 8
USP54	ubiquitin specific peptidase 54
VLDLR	very low density lipoprotein receptor
WIBG	within bgcn homolog (Drosophila)
WWC1	WW and C2 domain containing 1
XKR6	XK, Kell blood group complex subunit-related family, member 6
XYLB	xylulokinase homolog (H. influenzae)
YIPF1	Yip1 domain family, member 1
ZADH2	zinc binding alcohol dehydrogenase domain containing 2
ZBTB37	zinc finger and BTB domain containing 37
ZBTB7B	zinc finger and BTB domain containing 7B
ZDHHC11	zinc finger, DHHC-type containing 11
ZDHHC23	zinc finger, DHHC-type containing 23
ZMYND12	zinc finger, MYND-type containing 12
ZNF30	zinc finger protein 30
ZNF485	zinc finger protein 485
ZNF511	zinc finger protein 511
ZNF643	zinc finger protein 643
ZNF692	zinc finger protein 692
ZNF697	zinc finger protein 697
ZNF862	zinc finger protein 862

Suppl. Table 9: PCAT18 promoter analysis. From Chromosome 18 primary assembly (NC_000018.9) we downloaded 1Kb of 5`flanking sequence, immediately adjacent to the PCAT18 transcription start site. The sequence was uploaded in PROMO software (http://alggen.lsi.upc.es/cgi-bin/promo_v3/promo/promoinit.cgi?dirDB=TF_8.3), to identify human transcription factor binding sites (maximum dissimilarity: 5%). We identified 36 transcription factors, some with a known oncogenic role (c-Fos, c-Jun, STAT). The unique matrix that identifies each transcription factor is shown beside the official name. Notably, no AR binding site was detected. These findings were confirmed by analyzing a ChIP-on-chip dataset comprising AR binding sites in LNCaP cells exposed to castrate levels of androgens or 10nM DHT[23]. The authors identified a set of androgen-dependent and independent AR binding sites throughout the genome. Exploring these datasets, we found that the closest AR binding site is 29.9 Kb from the PCAT18 transcription starting site.

Transcription Factor	Matrix
AP-2alphaA	[T00035]
C/EBPalpha	[T00105]
C/EBPbeta	[T00581]
c-Ets-1	[T00112]
c-Ets-2	[T00113]
c-Fos	[T00123]
c-Jun	[T00133]
c-Myb	[T00137]
CTF	[T00174]
Elk-1	[T00250]
ER-alpha	[T00261]
FOXP3	[T04280]
GATA-1	[T00306]
GATA-2	[T00308]

GR	[T05076]
GR-alpha	[T00337]
GR-beta	[T01920]
HNF-1A	[T00368]
Ik-1	[T02702]
IRF-1	[T00423]
NF-AT1	[T01948]
NF-AT2	[T01945]
NF-Y	[T00150]
Pax-5	[T00070]
RelA	[T00594]
RXR-alpha	[T01345]
Sp1	[T00759]
STAT4	[T01577]
STAT5A	[T04683]
T3R-beta1	[T00851]
TBP	[T00794]
TFII-I	[T00824]
USF2	[T00878]
VDR	[T00885]
XBP-1	[T00902]
YY1	[T00915]
